# A Duplex qPCR Assay Targeting the *fadA* Gene Enables Robust Detection of *Fusobacterium* in Clinical Samples

**DOI:** 10.3390/ijms262311319

**Published:** 2025-11-23

**Authors:** Yurin Seo, Kyung-A Kim, Suho Lee, Yujin H. Lim, Yura Seo, Taeyul K. Kim, Chae Hyun Kim, Yeleem Kim, Francesca Jereis, Won Kyu Kim, Yoon Dae Han, Minsun Jung, Hyukmin Lee, Kyungwon Lee, Joong Bae Ahn, Jihoon G. Yoon, Han Sang Kim

**Affiliations:** 1 Yonsei Cancer Center, Division of Medical Oncology, Department of Internal Medicine, Yonsei University College of Medicine, Seoul 03722, Republic of Korea; kcs98949894@yonsei.ac.kr (Y.S.); hanulbory@yuhs.ac (K.-A.K.); 1002suho@naver.com (S.L.); yujinlim7@gmail.com (Y.H.L.); yura1029@yonsei.ac.kr (Y.S.); kimkj1731@yuhs.ac (T.K.K.); 2020162007@yonsei.ac.kr (C.H.K.); kimelie2374@gmail.com (Y.K.); vvswm513@yuhs.ac (J.B.A.); 2 Graduate School of Medical Science, Brain Korea 21 FOUR Project, Severance Biomedical Science Institute, Yonsei University College of Medicine, Seoul 03722, Republic of Korea; 3 School of Medicine, Georgetown University, Washington, DC 20007, USA; fj153@georgetown.edu; 4 Center for Natural Product Efficacy Optimization, Korea Institute of Science and Technology (KIST), Gangneung 25451, Republic of Korea; wkkim@kist.re.kr; 5 Division of Colon and Rectal Surgery, Department of Surgery, Yonsei University College of Medicine, Seoul 03722, Republic of Korea; acylyoon@yuhs.ac; 6 Department of Pathology, Yonsei University College of Medicine, Seoul 03722, Republic of Korea; jjunglammy@yuhs.ac; 7 Department of Laboratory Medicine, Yonsei University College of Medicine, Seoul 03722, Republic of Korea; hmlee71@yuhs.ac (H.L.); leekcp@yuhs.ac (K.L.); 8 Seoul Clinical Laboratories Academy, Yongin 16954, Republic of Korea; 9 Department of Laboratory Medicine, Gangnam Severance Hospital, Seoul 06273, Republic of Korea

**Keywords:** *Fusobacterium nucleatum*, *fadA*, qPCR, colorectal cancer, clinical detection

## Abstract

*Fusobacterium nucleatum* (*Fn*) is increasingly recognized as a cancer-associated bacterium, yet reliable quantification in human specimens is challenging due to low bacterial burden and abundant host DNA. We analyzed 145 *Fusobacterium* genomes to design primers targeting conserved regions of the *fadA* adhesin gene and developed a duplex quantitative real-time PCR (qPCR) assay for simultaneous detection of *fadA* and a human *PGT* as an internal control. Analytical sensitivity, specificity, precision, and reproducibility were evaluated using serially diluted *Fn* DNA, spike-in experiments with human DNA, and cross-platform/operator validation. Clinical performance was assessed in colorectal cancer patient tissues, including fresh tissue (*n* = 24) and formalin-fixed paraffin-embedded (FFPE) samples (*n* = 22), using 16S rRNA-based methods as references. The assay successfully detected all four major *Fn* subspecies (*nucleatum*, *animalis*, *polymorphum*, and *vincentii*). The limit of detection was ≤0.1 pg, with no interference between duplex targets. Spike-in experiments demonstrated consistent target detection in human-DNA-rich samples, with strong linearity (*R*^2^ = 0.998) across dilutions. High precision (coefficient of variations < 5%) was observed across intra-day, inter-day, inter-instrument, and inter-operator evaluations. In fresh tissues, the assay yielded 86% sensitivity, 94% specificity, and 92% accuracy. Using the FFPE samples, the assay achieved 91% sensitivity and 100% specificity, confirming robust classification in both clinical samples. This duplex qPCR assay enables broad detection of *Fn* with high analytical performance in both fresh and FFPE tissues. Its simplicity, reproducibility, and compatibility with pathology workflows support deployment in multi-center studies and downstream applications in diagnostic studies and prognostic modeling.

## 1. Introduction

*Fusobacterium nucleatum* (*Fn*) is an anaerobic, Gram-negative commensal bacterium that predominantly inhabits the human oral cavity, where it serves as a bridging organism through coaggregation with diverse microbes, thereby contributing to the development of chronic periodontitis [1,2]. Beyond oral disease, multiple studies have associated *Fn*’s role in cancer with immune evasion and adverse prognosis, including in colorectal, esophageal squamous cell, and head and neck malignancies [3,4,5,6,7,8]. Emerging evidence further links *Fn* abundance to response to anti-PD-1 immunotherapy, suggesting that *Fn* may modulate the tumor microenvironment and treatment sensitivity [9]. Multi-omics analyses have also delineated distinct *Fn* clades with colonization specific to tumor type patterns and differential virulence potential, providing mechanistic insight into its oncogenic involvement [10]. Collectively, these findings elevate *Fn* from a bystander commensal bacterium to a context-dependent pathogen that can influence cancer development, progression, and patient outcomes, highlighting the need for accurate detection and quantification in clinical specimens.

Several methods are used to detect *Fn* in tumors, each with advantages and limitations. RNA in situ hybridization visualizes the focal, pericellular distribution of *Fn* in colorectal cancer with single-molecule sensitivity and spatial resolution, but it is relatively costly and has low throughput [3,11]. Ex vivo anaerobic culture can recover viable organisms, yet positive culture rates are limited by the fastidious obligate anaerobic growth requirements of *Fn* [12]. Quantitative PCR (qPCR) enables high-throughput assessment of *Fn* DNA across large cohorts, supporting tumor enrichment relative to non-tumor controls and associations with poorer survival in colorectal, gastric, head and neck, and pancreatic cancers [4,8,13,14]. More recently, droplet digital PCR (ddPCR) has demonstrated improved limits of detection and reproducibility in formalin-fixed paraffin-embedded (FFPE) colorectal cancer tissues, and loop-mediated isothermal amplification has been utilized to target *Fn* via *fadA* [15,16,17]. Among these approaches, qPCR remains the most widely used in patient tissue studies [4,5,8,18,19].

In PCR-based approaches, common targets for detecting *Fusobacterium* include the 16S rRNA, *nusG*, and *fadA* genes [5,20,21,22,23,24]. The *nusG* gene, which encodes a transcription elongation factor, and the 16S rRNA gene are highly conserved within the *Fusobacterium* genus. These genes are widely used to assess both the presence and overall abundance of *Fusobacterium*. In contrast, FadA (Fusobacterium adhesin A, encoded by *fadA*) is a core adhesin largely restricted to the genus *Fusobacterium* that mediates epithelial adherence and invasion, contributing to inflammation and tumorigenic processes as a representative virulence factor. Multiple independent clinical and genomic studies have shown that colorectal cancer (CRC)-associated *Fusobacterium* lineages are predominantly composed of *fadA*-positive strains or those carrying homologues of *fadA*, and that *fadA*-positive *Fusobacterium* is detected more frequently in CRC tissues than in adjacent normal tissues or non-CRC subjects [23,25,26]. Accordingly, *fadA* is increasingly recognized as a valuable diagnostic target, given its high specificity for *Fusobacterium* and its direct clinical relevance to CRC.

A major limitation of current *fadA*-targeted qPCR assays is that sequence variability among *F. nucleatum* subspecies can result in varying detection sensitivity across the subspecies [10]. To address this, we analyzed publicly available *Fn* genomes to identify conserved regions within *fadA* and designed a new primer–probe set that robustly detects four major *F. nucleatum* subspecies, including *F. nucleatum* subsp. *nucleatum*, *animalis*, *polymorphum*, and *vincentii*. We demonstrate high analytical sensitivity, precision, and reproducibility, supporting the suitability of the assay for clinical and translational applications.

## 2. Results

### 2.1. Identification of Conserved Regions Within the fadA Gene Across Fusobacterium Genomes

**Figure 1:**
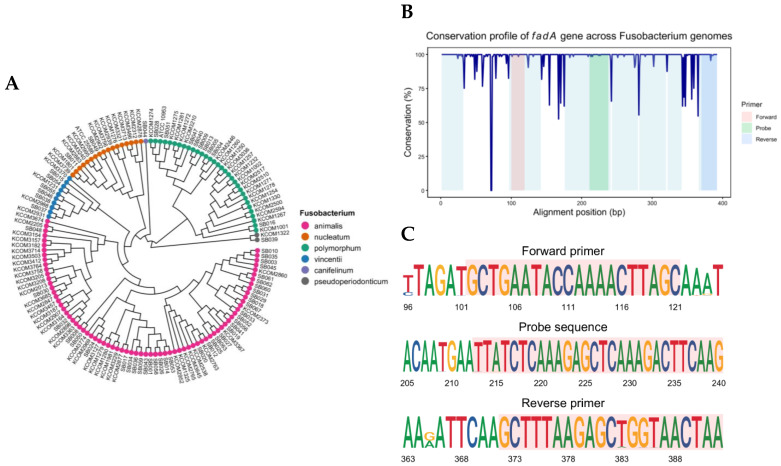
Comparative genomics of *Fusobacterium* and design of a conserved *fadA*-targeting qPCR assay. (**A**) Phylogenetic tree reconstructed from 137 *Fusobacterium* genomes harboring *fadA* (BioProject PRJNA549513). (**B**) Nucleotide conservation profile across the *fadA* locus. The y-axis indicates percent identity at each aligned position. Shaded bands mark the regions selected for the forward primer (pink), probe (green), and reverse primer (blue). (**C**) Sequence logos for the primer and probe target regions showing base frequencies and information content. Red-shaded bands highlight the primer sequences. High conservation across these regions supports the use of a cross-subspecies primer–probe design for the broad detection of *fadA*.

To comprehensively assess the genetic diversity across the genus, we analyzed 145 publicly available *Fusobacterium* genomes retrieved from the National Center for Biotechnology Information (NCBI) database (Supplementary Table S1). Phylogenetic reconstruction revealed distinct clades corresponding to major *Fusobacterium* species, including *Fn* subspecies (*nucleatum*, *animalis*, *polymorphum*, and *vincentii*; Figure 1A).

Given the central role of *fadA* in adhesion and host-pathogen interactions, we profiled nucleotide conservation across the gene. We observed that all 134 *Fn* genomes harbored *fadA,* and certain non-*nucleatum* species, such as *F.canifelinum* and *F.pseudoperiodonticum*, also possessed *fadA* homologs with >90% sequence identity. In contrast, eight non-*nucleatum* strains, including *F. necrophorum*, *F. ulcerans*, and *F. varium*, either lacked *fadA* entirely or contained only low-homology sequences. Multiple segments showed high conservation (≥95% identity) across the majority of genomes (Figure 1B). We prioritized these windows for primer–probe design to maximize cross-subspecies coverage. The final assay amplifies a 291-bp region within *fadA*, with in silico coverage across all 137 genomes harboring the gene. Based on these findings, we established a duplex qPCR targeting *fadA* with the human *PGT* gene as the internal control.

### 2.2. Assessment of the Duplex qPCR Assay

#### 2.2.1. Cross-Target Interference

**Figure 2:**
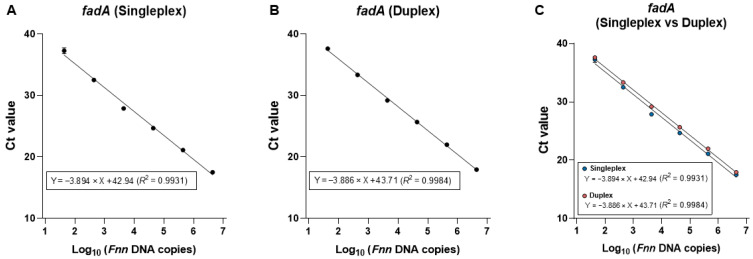
Cross-target interference was assessed by comparing standard curves for *fadA* detection in singleplex and duplex qPCR. *Fnn* DNA was serially diluted from 10 to 10^−4^ ng (4.35 × 10^6^ to 43.5 genome copies per reaction). (**A**) Singleplex qPCR for *fadA* and (**B**) duplex qPCR for *fadA* and *PGT* were performed. (**C**) The comparison showed nearly identical standard curves, indicating minimal cross-target interference. Experiments were performed in triplicate.

To test whether multiplexing affects performance, we compared singleplex (*fadA* only) and duplex (*fadA* + human *PGT* internal control) reactions using genomic DNA from *Fn* subspecies nucleatum (*Fnn*, ATCC 25586), serially diluted 10-fold from 10 to 10^−4^ ng. In the singleplex assay, only *fadA* was amplified, while duplex qPCR was conducted using both *fadA* and *PGT* primers (Figure 2A,B). *PGT* amplification efficiency has been previously validated [4]. Standard curves were nearly superimposable, with comparable slopes (−3.86 vs. −3.89) and similar amplification efficiencies (~81–83%) across the dilution series (Figure 2C). Thus, inclusion of the *PGT* internal control does not measurably affect *fadA* amplification efficiency or dynamic range, indicating no primer-probe interference within the duplex qPCR assay.

#### 2.2.2. Analytical Sensitivity

**Figure 3:**
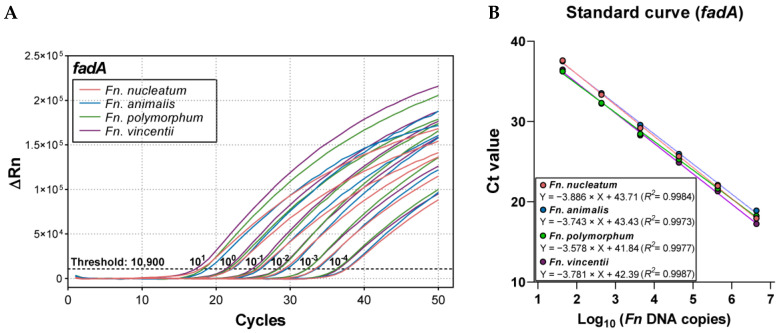
Sensitivity of the *fadA* Duplex qPCR assay. (**A**) Amplification curves for the four *Fusobacterium nucleatum* (*Fn*) subspecies: *Fn. nucleatum*, *Fn. animalis*, *Fn. polymorphum*, and *Fn. vincentii*, from gDNA dilutions of 10 to 10^−4^ ng (4.35 × 10^6^ to 43.5 genome copies per reaction). (**B**) Standard curves showing the relationship between Ct values and log_10_ of DNA copies for each subspecies. Experiments were performed in triplicate.

Next, to determine the analytical sensitivity of the duplex qPCR, genomic DNA samples from four *Fn* subspecies, including *Fnn*, *Fn animalis* (*Fna*; ATCC 51191), *Fn polymorphum* (*Fnp*; ATCC 10953), and *Fn vincentii* (*Fnv*; ATCC 49256), were serially diluted 10-fold from 10 to 10^−4^ ng per reaction (approximately 4.35 × 10^6^ to 43.5 genome copies per reaction). Robust amplification was observed across the complete five-log dilution series for all strains (Figure 3A). Standard curves demonstrated a high linear correlation between Ct and log_10_ input copies (*R*^2^ ≥ 0.997), with slopes ranging from −3.578 to −3.886, corresponding to amplification efficiencies of approximately 90% (Figure 3B). The limit of detection (LOD) was 0.1 pg (approximately 43.5 copies per reaction) across all four subspecies.

#### 2.2.3. Analytical Specificity

**Figure 4:**
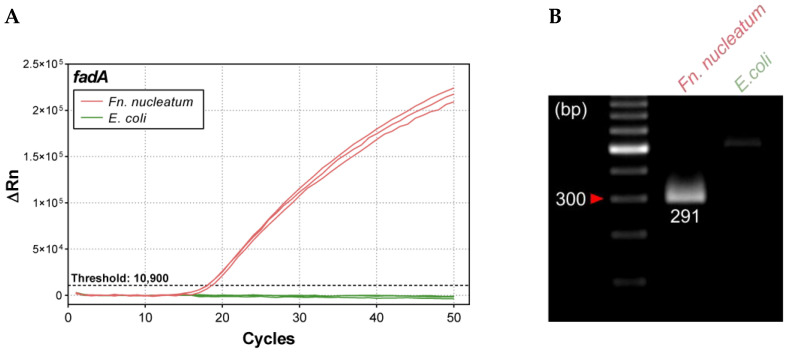
Specificity of the *fadA* Duplex qPCR Assay. *Fn. nucleatum* was the positive control, and *E. coli* was the negative control. (**A**) Amplification curves for 10 ng of each organism. Experiments were performed in triplicate. (**B**) Agarose gel electrophoresis showing a 291-bp product for *Fn. nucleatum* and no amplification for *E. coli*.

We assessed specificity using genomic DNA from *Fnn* (positive control) and *E. coli* MG1655 (ATCC 47076; negative control). The *fadA*-specific probe yielded robust amplification exclusively with *Fnn* DNA, with no amplification from *E. coli* (Figure 4A). Consistently, agarose gel electrophoresis showed a single 291-bp product only in the *Fnn* reaction, matching the expected *fadA* amplicon target (Figure 4B). These data demonstrate that the duplex qPCR assay specifically detects the *fadA* gene of *Fn* without cross-reactivity to non-target bacterial DNA under the tested conditions.

#### 2.2.4. Robust Detection of Fusobacterium DNA in a Human Genomic Background

**Figure 5:**
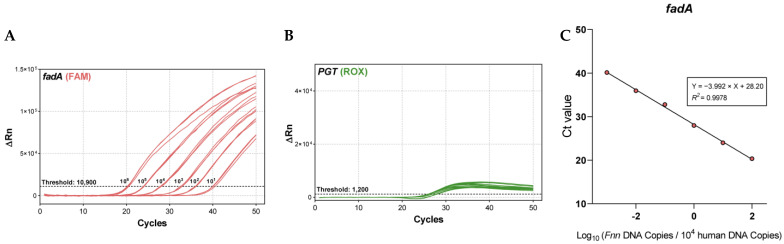
Spike-in test to mimic human-derived samples. A constant amount of human reference DNA (10,000 copies) was combined with *Fnn* DNA, which was serially diluted from 1,000,000 to 10 copies. (**A**) Amplification curves for *fadA*. (**B**) Amplification curves for *PGT*. (**C**) Standard curves showing the relationship between Ct values and log_10_ (*Fnn* DNA copies/10^4^ human DNA copies). Experiments were performed in triplicate.

To evaluate performance in a host-DNA-rich matrix, we performed spike-in experiments by serially diluting Fnn genomic DNA 10-fold across six steps (10^6^ to 10 copies per reaction) and mixing each dilution with 10^4^ diploid genome equivalents (approximately 66 ng) of human reference DNA (NA12155). This produced human: Fnn ratios from 1:100 to 1000:1. As expected, the *fadA* channel showed stepwise Ct increases with decreasing Fnn input (Figure 5A), whereas the *PGT* internal control amplified stably across all mixtures (Figure 5B). Standard-curve analysis demonstrated excellent linearity between Ct and log_10_ (Fnn copies per 10^4^ human genome copies) (*R*^2^ = 0.998), with a slope of −3.992 corresponding to an amplification efficiency of ~78% (Figure 5C).

Together, these results indicate minimal matrix effects from excess human DNA and confirm that the duplex qPCR reliably detects *Fnn* down to 10 copies per reaction while maintaining consistent internal control performance, supporting applicability to complex human-derived samples.

#### 2.2.5. Precision and Reproducibility

To assess the repeatability of the duplex qPCR, intra-day, inter-day, inter-instrument, and inter-operator variations in Ct values were evaluated for serial dilutions of *Fnn* DNA (from 10 ng to 10^−4^ ng) (Table 1). For the intra-day test, three independent runs were performed on the same day. The inter-day test was conducted by repeating the same assay on three separate days at two-day intervals. For inter-instrument evaluation, two qPCR platforms, StepOne Plus and QuantStudio 3, were tested. For inter-operator reproducibility, three independent operators performed the assay under identical conditions.

The results showed that the intra-day coefficient of variation (CV) ranged from 0.2% to 1.4%, the inter-day CV ranged from 1.3% to 3.5%, the inter-instrument CV ranged from 1.1% to 4.6%, and the inter-operator CV ranged from 0.7% to 2.5%. All Ct values had a CV of less than 5%, which demonstrates that this detection method has excellent stability and reproducibility.

### 2.3. Clinical Validation and ΔCt Cutoff Determination of the Duplex qPCR

To establish a ΔCt cutoff and evaluate the diagnostic performance of the *fadA* duplex qPCR assay, we analyzed 24 fresh-tissue tumor samples and 22 FFPE colorectal cancer specimens (Supplementary Table S2). We used 16S rRNA sequencing as the reference method.

**Figure 6:**
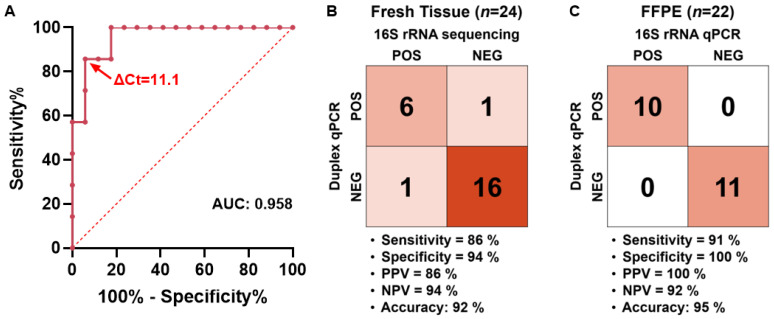
Clinical validation of the *fadA* duplex qPCR assay. (**A**) Receiver Operating Characteristic (ROC) curve for fresh colorectal cancer tissues (*n* = 24), generated using ΔCt values from the *fadA* duplex qPCR, with 16S rRNA sequencing as the reference standard (AUC = 0.958. The red arrow indicates the operational cut-off (ΔCt = 11.1), defined as the median ΔCt within the range of 10.4 to 11.8, where the Youden index reaches its maximum value (J = 0.798). (**B**) Confusion matrix comparing 16S rRNA sequencing and the duplex qPCR in fresh tissues (*n* = 24) at the operational cutoff of ΔCt = 11.1. (**C**) Confusion matrix comparing 16S rRNA qPCR and the duplex qPCR in formalin-fixed paraffin-embedded (FFPE) samples (*n* = 22) using the same cutoff. Sensitivity, specificity, positive predictive value (PPV), negative predictive value (NPV), and accuracy are indicated for each matrix.

In fresh tissues, *Fusobacterium* positivity was determined by the 16S rRNA sequencing method. The ΔCt values from the corresponding *fadA* duplex qPCR were then used to construct a receiver operating characteristic (ROC) curve to determine the optimal ΔCt cutoff (Figure 6A). The area under the curve (AUC) was 0.958. The Youden index reached its highest value of 0.798 within the ΔCt range of 10.4 to 11.8. Consequently, the median of this interval, ΔCt = 11.1, was chosen as the operational cutoff. At this cutoff, the duplex qPCR showed a sensitivity of 86% (95% confidence interval [CI], 48.69–99.27%) and a specificity of 94% (95% CI, 73.02–99.70%), with a positive predictive value (PPV) of 86%, a negative predictive value (NPV) of 94%, and an overall accuracy of 92% (Figure 6B).

We then applied the same ΔCt cutoff to the 22 FFPE specimens and benchmarked the results against those obtained with conventional 16S primer-based qPCR [4]. The duplex qPCR exhibited equivalent specificity (100%) but a slightly lower sensitivity (91%), which was expected given that the assay selectively targets *Fusobacterium* strains harboring the *fadA* gene (Figure 6C).

Collectively, these data support the duplex qPCR as a robust and specific method for detecting *Fusobacterium* across fresh and FFPE clinical samples, with a simple ΔCt-based rule that performs well against established 16S-based assays.

## 3. Discussion

In this study, we developed and validated a duplex qPCR assay targeting *fadA* for the reliable detection of cancer-associated Fusobacterium strains in clinical specimens. Through comparative genomic analysis of large-scale and publicly available *Fusobacterium* genomes, we identified highly conserved *fadA* regions and designed primers optimized for broad coverage across subspecies. This genomics-informed design minimizes strain-dependent variability and enhances the assay’s sensitivity for diverse *Fn* lineages. The assay demonstrated consistent detection of the four major *Fn* subspecies (*Fnn*, *Fna*, *Fnp*, and *Fnv*), with robust performance maintained even in host DNA-rich conditions. Although experimental validation for minor subspecies was not conducted, our in silico analyses suggest that the assay could also detect related subspecies such as *F. canifelium* and *F.pseudoperiodonticum*. The high precision and reproducibility observed in various conditions suggest that the developed assay may be suitable for application in multi-center and large-cohort studies.

Prior PCR-based approaches have commonly targeted 16S rRNA, *nusG*, or virulence factors such as *fadA* [5,20,21,22,23,24]. Unlike 16S rRNA or *nusG*, *fadA* can exhibit subspecies sequence diversity [10], which risks uneven detection. By investigating large-scale genomes to identify conserved regions within *fadA*, we achieved comparable analytical sensitivity across the four subspecies tested. To our knowledge, this issue has not been systematically examined in previous studies.

We developed a duplex qPCR assay that allows for the simultaneous quantification of the *fadA* gene and a human reference target within a single reaction. This approach enhances the efficiency of using limited patient samples and increases throughput by reducing the number of necessary reactions and the hands-on time involved. In FFPE samples, where DNA is often fragmented, ddPCR may provide higher analytical sensitivity. However, the duplex qPCR assay can be conducted on standard real-time PCR instruments that are commonly available in most diagnostic laboratories. This makes it easy to integrate into routine pathology and molecular workflows, presenting a practical option for large-cohort studies and potential future clinical applications.

The further development of the assay could involve multiplexing with additional *Fusobacterium*-related targets. For instance, combining the current assay with targets such as the 16S rRNA gene or *nusG* would allow for the simultaneous detection of *fadA*-positive, CRC-associated strains, as well as pan-*Fusobacterium* signals within a single reaction. A panel of target-specific primer-probe sets would help to broaden the detection range while maintaining assay specificity. Additionally, developing a multiplex qPCR panel that includes *fadA* along with other virulence- and colonization-associated genes could be used to investigate the relationships between specific combinations of virulence factors and clinical characteristics.

The limitations of our study include the modest size of clinical cohorts from which the tissue samples were derived. As the proposed ΔCt cutoff was established from this cohort, external validations in large and more diverse sample sets will be necessary to assess generalizability across platforms, laboratories, and extraction protocols. In fresh tissue samples, the false negative (ΔCt = 12.2) and false positive (ΔCt = 9.6) values are considered borderline cases, as they are close to the predetermined ΔCt cut-off. Increasing the sample size and implementing a three-tier reporting scheme, such as definite positive, indeterminate (recommended for retesting), and negative, could enhance diagnostic accuracy.

Additionally, the performance of the assay in non-tissue specimens such as stool or saliva was not evaluated in this study. This assay was optimized with the assumption of a low bacterial load and a high background of human DNA. However, in specimens like stool or saliva, where the total bacterial burden and PCR inhibitors can vary significantly, it may be necessary to determine separate cutoffs or conduct additional validation. In liquid biopsy samples such as plasma or serum, the amount of bacterial DNA is likely to be even lower, so specialized pre-analytical processing may be required. Establishing its applicability in such non-invasive specimens will be particularly important for clinical application in early screening and long-term monitoring [27,28].

This study evaluated the performance of a duplex qPCR assay on clinical colorectal cancer tissues (fresh and FFPE) using 16S rRNA sequencing as the reference standard. Although shotgun metagenomic sequencing could offer a more accurate reference, it was impractical due to severe DNA fragmentation in FFPE samples, low bacterial burden in fresh tissues, and associated costs. 16S rRNA-based methods have limitations, including variations in 16S copy number and primer-binding efficiency among *Fusobacterium* subspecies, which can result in amplification bias and variable sensitivity. Additionally, low-biomass samples may lead to contamination or amplification failures, causing false-positive or false-negative results. Therefore, the sensitivity and specificity reported here reflect the potential biases of the 16S rRNA-based reference and should be interpreted accordingly.

Our assay showed lower sensitivity compared to specificity. This is likely due to the reference standard (16S rRNA sequencing) featuring fewer positive samples (*n* = 7) than negative samples (*n* = 17), leading to a conservative estimate of sensitivity. In FFPE samples, the false negative had a ΔCt of 20.2 (Ct*_fadA_* = 48.7, Ct*_PGT_* = 28.5). Because our assay specifically targets *fadA*-harboring *Fusobacterium* strains, certain taxa, such as *F. necrophorum*, *F. ulcerans*, and *F. varium*, which are 16S-positive but *fadA*-negative, may go undetected, which could further lower the apparent sensitivity of the assay. Although the association between colorectal cancer and *F. necrophorum* remains unclear, the reported enrichment of *F. ulcerans* and *F. varium* in Southern Chinese individuals with colorectal cancer highlights the need for future refinement of the assay to ensure broader taxonomic coverage [26]. Finally, pre-analytical variables, such as fixation time, tumor cellularity, DNA fragmentation in FFPE samples, as well as biological variation in the abundance of the human reference target, could influence ΔCt values and should be systematically evaluated in future studies.

In conclusion, we developed and validated a genomics-informed duplex qPCR assay that enables broad, subspecies-inclusive detection of *Fn* with high reproducibility and applicability in both fresh and FFPE tissues. With further external validation in various cohorts and specimens, standard operating procedures, and potential integration into multiplex platforms, this assay could serve as a robust tool for prognostic modeling and for elucidating the clinical relevance of *Fusobacterium* in cancer patients.

## 4. Materials and Methods

### 4.1. Acquisition of Fusobacterium Genomes

The genomic data of publicly available *Fusobacterium*, derived from oral cavity samples and colorectal cancer tumor tissues, were retrieved from the NCBI database under BioProject accession number PRJNA549513 (https://www.ncbi.nlm.nih.gov/bioproject/PRJNA549513/ (accessed on 22 January 2025)) [10]. The genomic data, obtained via the NCBI datasets command-line tool (ncbi-datasets-cli), included assemblies generated through single-molecule real-time sequencing. Genome files were stored locally in FNA format for downstream computational analyses. *Fusobacterium nucleatum* subsp. *nucleatum* (ATCC 25586) was selected as the reference genome to facilitate comparative analyses. We targeted adhesion protein *fadA* (Gene ID: 79782470) for fusobacterial detection, and the sequence was obtained from the NCBI in a FASTA format for downstream analyses.

### 4.2. Primer and Probe Design

By analyzing 145 *Fusobacterium* genomes, we identified highly conserved sequences of the *fadA* gene (Supplementary Table S1). We utilized the Basic Local Alignment Search Tool (BLAST) v2.16.0 [29], and each genome was aligned against the *fadA* gene sequences of the reference genome using the *blastn* command with an e-value threshold of 10^−5^. The BLAST output was formatted to display key alignment metrics, including query coverage, percent identity, alignment length, and bit score (output format 6). For each genome, the top alignment based on bit score and alignment length was extracted using custom scripts. Extracted sequences were saved as individual FASTA files, ensuring the removal of redundant or low-quality hits. Gene sequences identified by BLAST were merged into a single FASTA file for each target gene and aligned using Clustal Omega v1.2.4 (*clustalo*) [30]. The alignments were manually inspected using *Jalview* v2.11.50 to ensure the integrity and quality of conserved regions [31]. Gaps and low-confidence regions were removed using sequence-specific filters. Consensus sequences with at least 95% conservation across genomes were selected for primer and probe design. PCR primers were designed with criteria specifying a primer length between 18 and 25 base pairs, a melting temperature (Tm) between 54 and 58 °C, and a product size ranging from 100 to 300 base pairs. Probes for quantitative PCR were designed within the conserved regions with specifications that included a probe length between 20 and 30 base pairs and a Tm between 64 and 68 °C. Additionally, we utilized the human prostaglandin transporter (*PGT*; *SLCO2A1*) gene as an internal control, as described previously [4]. The *PGT* reference gene is a single-copy autosomal gene on chromosome 3 and was used as the internal reference for determining human gene copy number [32]. This locus has been widely adopted as a human reference gene in qPCR assays quantifying *Fn* in fresh and FFPE colorectal tissue samples from CRC patients [4,33,34]. By targeting both *fadA* and *PGT*, we established a duplex qPCR system to correct for differences in DNA input and quality across specimens and to reduce variability within a single reaction, thereby improving measurement reliability. All designed primers and probes were further evaluated in silico for specificity using BLAST against the *Fusobacterium* genomes and screened for secondary structures, including hairpins and primer-dimerization, to ensure optimal amplification and detection. The corresponding primer and probe sequences are listed in Table 2. All primers and probes were synthesized commercially (Macrogen, Seoul, Republic of Korea).

### 4.3. Bacterial Strains

*Fusobacterium nucleatum* subsp. *nucleatum* (Fnn) (ATCC 25586), *Fusobacterium nucleatum* subsp. *animalis* (ATCC 51191), *Fusobacterium nucleatum* subsp. *polymorphum* (Fnp) (ATCC 10953), and *Fusobacterium nucleatum* subsp. *vincentii* (Fnv) (ATCC 49256) were purchased from the American Type Culture Collection (ATCC, Manassas, VA, USA). *Escherichia coli* (*E. coli*) (ATCC 47076) was obtained from the Korean Culture Center of Microorganisms (KCCM, Seoul, Republic of Korea).

*Fusobacterium* strains were cultured on Brucella agar plates supplemented with 5% sheep blood and incubated anaerobically at 37 °C for 48–72 h in a Coy anaerobic chamber (Coy Laboratory Product, Grass Lake, MI, USA) under a gas mixture of 20% CO_2_, 5% H_2_, and 75% N_2_. Colonies were picked and inoculated into a Gifu Anaerobic Medium (GAM) (KisanBio, Seoul, Republic of Korea) broth and incubated for 18 h. Cells were harvested by centrifugation at 3000× *g* for 10 min and washed twice with phosphate-buffered saline (PBS).

*E. coli* (ATCC 47076) was cultured on Luria-Bertani (LB) agar (Difco, New York, NY, USA) plate and incubated aerobically at 37 °C for 24 h. Colonies were inoculated into LB broth (Difco, USA) and incubated for 18 h. Cells were harvested by centrifugation at 3000× *g* for 10 min and washed twice with PBS.

### 4.4. DNA Extraction Methods

DNA was extracted using the QIAamp DNA Mini Kit (Qiagen, Crawley, UK). For bacterial pellets or surgically fresh tissues, the samples were suspended with protease K in ATL buffer and incubated at 56 °C for 2 h. Both AL buffer and absolute ethanol were added to the samples before applying the QIAamp spin column. Each sample was centrifuged and washed according to the manufacturer’s protocol. DNA was eluted from the column with 50 μL of the supplied AE buffer.

DNA extraction from FFPE tissue sections (10 μm thickness) was performed using the QIAamp DNA FFPE Tissue Kit according to the manufacturer’s instructions. Briefly, the paraffin was removed with 1 mL xylene followed by a wash in 96–100% ethanol. The pellet was resuspended in ATL buffer containing proteinase K and incubated at 56 °C for at least 2 h. After lysis, the subsequent clean-up steps included incubation at 90 °C to reverse formaldehyde-induced crosslinking, treatment with RNase A, ethanol precipitation, DNA binding to a MinElute column (Qiagen), and washing. Finally, the DNA was eluted in 55 μL elution buffer. Quality and quantity of the isolated DNA were determined using a NanoDrop spectrophotometer (ND-1000; Thermo Scientific, Waltham, MA, USA).

### 4.5. Quantitative Polymerase Chain Reaction (qPCR)

Quantitative Polymerase Chain Reaction (qPCR) was performed using a hydrolysis probe-based (TaqMan) chemistry in a 20 µL reaction mixture containing 1× TaqPath™ ProAmp™ Multiplex Master Mix (Applied Biosystems, San Francisco, CA, USA), 500 nM of each primer, 300 nM of each probe, nuclease-free water, and template DNA. For spike-in tests and clinical validation using fresh tissue or FFPE samples, we used *fadA* primers at a concentration of 900 nM for each primer, along with probes at 250 nM to enhance the efficiency of *Fn* detection. Additionally, *PGT* primers were used at a concentration of 150 nM for each primer, accompanied by probes at a concentration of 250 nM. Amplification was conducted on a StepOnePlus or QuantStudio 3 Real-Time PCR system (Applied Biosystems, USA) with the following cycling conditions: initial denaturation at 95 °C for 10 min, followed by 50 cycles of 95 °C for 15 s and 60 °C for 1 min. The cycle threshold (Ct) was determined by manually adjusting the threshold, defined as the ΔRn value at which the fluorescence signal is considered significantly above the baseline. When testing with strain DNA, experiments were conducted on a single plate to eliminate any differences between batches. In this case, the threshold for *fadA* was set at 10,900, while *PGT* was set at 1200. For clinical samples, however, different batches were used, so batch differences needed to be accounted for. Therefore, the *fadA* threshold was set as 1.2% of the maximum ΔRn value from each experiment, and the *PGT* threshold was set at 4.4%. ΔCt was calculated as Ct*_fadA_*–Ct*_PGT_*, as described previously [4]. All experiments were conducted in triplicate to ensure reproducibility.

### 4.6. 16S rRNA Sequencing

The *Fn* positivity in primary tumor tissue samples from colorectal cancer (CRC) patients was determined through 16S rRNA sequencing in a previous study [36] (Supplementary Table S2). DNA concentration from surgical tumor tissues was measured with the Qubit dsDNA HS Assay Kit and a Qubit Fluorometer (Invitrogen, Carlsbad, CA, USA). Library preparation for 16S rRNA gene sequencing followed the Illumina protocol. Amplicon PCR targeting the bacterial 16S rRNA V3-V4 region (primers Bakt_341F-805R) was performed under the following conditions: 95 °C for 3 min; 25 cycles of 95 °C for 30 s, 55 °C for 30 s, 72 °C for 30 s; final extension at 72 °C for 5 min. PCR products were verified on an Agilent Bioanalyzer (DNA 1000 chip, Santa Clara, CA, USA) and quantified using a Qubit Fluorometer. Amplicons were purified using AMPure XP beads (Beckman Coulter, Brea, CA, USA), followed by index PCR with Nextera^®^ XT Index primers (Illumina, San Diego, CA, USA) under the same cycling conditions, except for 8 cycles. Indexed libraries were purified with AMPure XP beads, and library concentration was determined using the Quant-iT™ PicoGreen™ dsDNA Assay Kit (Invitrogen, USA). Sequencing was performed on the Illumina MiSeq platform (Macrogen, Republic of Korea), generating 2 × 300 bp paired-end reads.

Raw paired-end reads were assembled using FLASH (v1.2.11) [37] to merge overlapping sequences and improve quality. Assembled reads were length filtered with CD-HIT-OUT (v4.8.1) [38], retaining sequences between 400–500 bp. Redundant sequences were clustered at 100% identity using CD-HIT-DUP, and chimeric sequences were removed. Secondary clusters were merged into primary clusters, and noise sequences were filtered out based on size thresholds. Non-chimeric representative reads were clustered into OTUs at 97% identity for species-level classification using a greedy algorithm. Taxonomic assignment of OTUs was performed with QIIME against the NCBI 16S rRNA database (version 20211127). Taxonomic abundance ratios were calculated, and samples were classified as *Fn*-positive if *Fn* exceeded 1% relative abundance.

### 4.7. DNA Gel Electrophoresis

Amplified qPCR products were mixed with 6× loading buffer (Dyne Bio, Seongnam-si, Republic of Korea) and subjected to electrophoresis on a 2% agarose gel prepared in 0.5× Tris-acetate-EDTA (TAE) buffer. The gel was supplemented with nucleic acid staining solution (Dyne Bio, Republic of Korea), and electrophoresis was carried out at 100 V for 30 min. DNA bands were visualized and documented using a MINIBIS Pro Gel Documentation System (DNR, Jerusalem, Israel).

### 4.8. Determination of Sensitivity and Specificity

Linearity was evaluated following the Clinical and Laboratory Standards Institute guideline EP06 [39]. The sensitivity of *fadA* detection was evaluated using genomic DNA from *Fnn*, serially diluted 10-fold from 10^1^ to 10^−4^ ng. Amplification efficiency was assessed by both singleplex (*fadA* only) and duplex (*fadA* + *PGT*) qPCR. Standard curves were generated from Ct values, and the slope and coefficient of determination (R^2^) were calculated by linear regression. The limit of detection (LOD) was assessed based on the minimum copy number detectable within 40 amplification cycles. The specificity of *fadA* detection was assessed using 10 ng of *Fnn* DNA as a positive control and genomic DNA from *E. coli* as a negative control.

### 4.9. Spike-In Test

Assuming 6.6 pg of DNA per diploid human genome, 66 ng of genomic DNA represents 10^4^ copies of the human genome. Considering that the genome size of *Fnn* (ATCC 25586) is approximately 2.2 Mb, and that a single genome weighs 2.38 fg (1 × 10^−15^ g), 10^6^ genomic copies correspond to 2.3 ng of *Fnn* DNA. To mimic human tissue colonized by *Fnn*, human genomic DNA (NA12155; 66 ng, approximately 1 × 10^4^ diploid genome equivalents) was mixed with *Fnn* genomic DNA, serially diluted in a 10-fold manner across six steps (from 10^6^ to 10^1^ copies per reaction). Amplification was assessed by duplex qPCR targeting *fadA* and *PGT*. Standard curves were generated from Ct values, and slope and *R*^2^ were calculated by linear regression.

### 4.10. Stability and Reproducibility

The stability and reproducibility of the assay were evaluated using *Fnn* DNA serially diluted 10-fold across six concentrations (10^1^ to 10^−4^ ng). The study design and statistical analysis were based on the Clinical and Laboratory Standards Institute guideline EP15-A3 [40]. Intra-day variation was determined by performing three independent replicates within a single day, whereas inter-day variation was assessed by repeating the assay on three separate days at two-day intervals. The standard deviation (SD) and coefficient of variation (CV) were calculated from the mean Ct values of triplicate reactions.

### 4.11. Statistical Analysis

To evaluate the clinical performance of the duplex qPCR assay, receiver operating characteristic (ROC) analysis was performed, and the area under the curve (AUC) was calculated using GraphPad Prism (v8.0.1), with 16S rRNA-based detection as the reference standard. The Youden index was calculated as sensitivity plus specificity minus one, and 95% confidence intervals for sensitivity and specificity at the chosen cutoff were obtained using the Wilson–Brown method.

### 4.12. Ethical Approval

This study received approval from the Institutional Review Board (IRB) of Yonsei University Severance Hospital, under IRB No. 4-2019-0811. Before enrollment and sample collection, written informed consent was obtained from all participants at Yonsei University College of Medicine, Severance Hospital. The research adhered to the principles outlined in the Declaration of Helsinki.

## 5. Patents

US/63/814,468, Pending: Composition for detecting genus Fusobacterium and uses thereof, Inventor: Han Sang Kim (2025).

## Figures and Tables

**Table 1:** Ct values and precision metrics (CV%) of the duplex qPCR assay across intra-day, inter-day, inter-instrumental, and inter-operator assessments.

Detection Target	*Fnn* DNA (ng)	Intra-Day		Inter-Day		Inter-Instrument		Inter-Operator	
		Ct	CV (%) *	Ct	CV (%) *	Ct	CV (%) *	Ct	CV (%) *
*fadA*	10^1^	18.0 ± 0.1	0.8	17.8 ± 0.2	1.3	17.5 ± 0.6	3.3	17.5 ± 0.4	2.5
	10^0^	21.9 ± 0.1	0.6	21.6 ± 0.7	3.1	21.3 ± 1.0	4.5	21.0 ± 0.2	1.1
	10^−1^	25.6 ± 0.1	0.2	25.2 ± 0.7	2.7	25.9 ± 0.3	1.1	24.8 ± 0.4	1.6
	10^−2^	29.1 ± 0.1	0.5	28.7 ± 0.6	2.2	30.2 ± 1.4	4.6	28.4 ± 0.5	1.7
	10^−3^	33.2 ± 0.2	0.5	32.7 ± 0.7	2.1	34.1 ± 1.1	3.2	32.3 ± 0.3	1.0
	10^−4^	37.0 ± 0.5	1.4	37.2 ± 1.3	3.5	38.4 ± 1.0	2.7	36.0 ± 0.3	0.7

* CV coefficient of variation; calculated as (standard deviation of Ct/mean Ct) × 100.

**Table 2:** Primer and probe sequences used for quantitative PCR.

Target Gene	Name	Sequence (5′-3′)	Reference
*fadA*	*fadA*-Forward	GCTGAATACCAAAACTTAGC	this study
	*fadA*-Reverse	TTAGTTACCAGCTCTTAAAGC	
	*fadA*-Probe	(FAM)-TTATCTCAAAGAGCTCAAAGACTTCAAG-(BHQ1)	
*PGT*	*PGT*-Forward	ATCCCCAAAGCACCTGGTTT	[4]
	*PGT*-Reverse	AGAGGCCAAGATAGTCCTGGTAA	
	*PGT*-Probe-ROX	(ROX)-CCATCCATGTCCTCATCTC-(BHQ2)	
	*PGT*-Probe-FAM	(FAM)-CCATCCATGTCCTCATCTC-(TAMRA)	
16S rRNA	16S-Forward	GGATTTATTGGGCGTAAAGC	[35]
	16S-Reverse	GGCATTCCTACAAATATCTACGAA	
	16S-Probe	(FAM)-CTCTACACTTGTAGTTCCG-(TAMRA)	

## Data Availability

The original contributions presented in this study are included in the article/Supplementary Materials. Further inquiries can be directed to the corresponding authors.

## Supplementary Materials

The following supporting information can be downloaded at: https://www.mdpi.com/article/10.3390/ijms262311319/s1.
